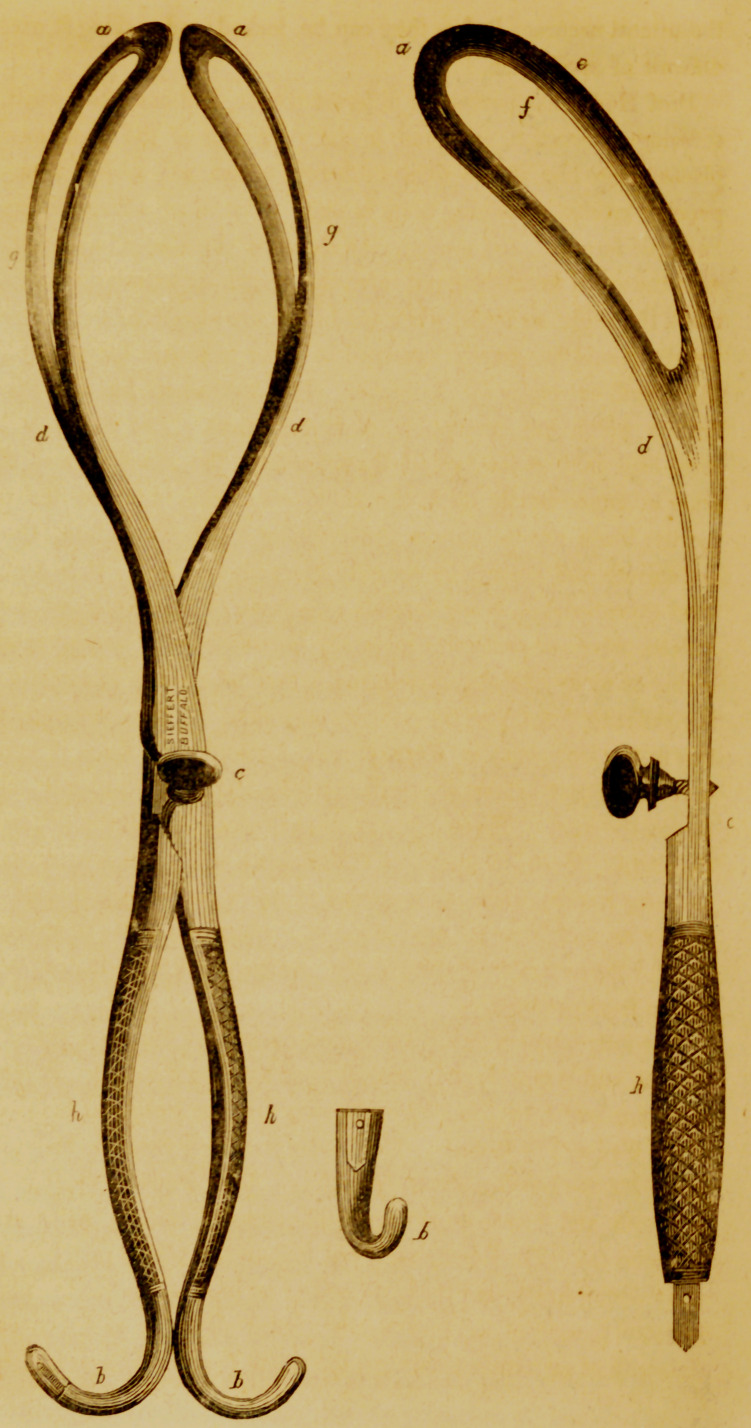# Remarks on the Construction of Obstetrical Forceps, with a Description of an Instrument

**Published:** 1849-05

**Authors:** James P. White

**Affiliations:** Prof of Obstetrics in Med. Dep’t University of Buffalo


// BUFFALO MEDICAL JOURNAL

AND

MONTHLY REVIEW.

VOL. 4. MAY, 1849. NO. 12.

### ORIGINAL COMMUNICATIONS.

ART. I.—*Remarks on the Construction of Obstetrical Forceps, with a Description of an Instrument, Employed by* James P. White, M. D. Prof. of Obstetrics in Med. Dep't University of Buffalo.

Mr Editor:—

In the belief that the most useful of all obstetric instruments, the forceps, is exceedingly defective in construction, as we find it in the hands of most practitioners in this country, I hope to render a service, not entirely unacceptable, by calling the attention of the profession to the subject. I trust, also, that the instrument which I shall recommend will be found to combine all the advantages possessed by many of the various instruments, used by different accoucheurs, in this country and in Europe.

In addition to this eclecticism, I flatter myself that I have been able to make a few suggestions, not altogether unworthy of consideration. Should I fail in the undertaking, may I not at least hope, that sufficient interest will be awakened, to induce some more ingenious observer to direct his attention to the subject, who will substitute a safer and better instrument than is now offered for sale by most of the makers and venders in this country?

There can be little doubt that much of the discredit cast upon its application in suitable cases, has arisen from the fact that the instrument used was not adapted to fulfill all the purposes for which it was designed. Either it was too heavy and thick; it did not possess the requisite length and curves to seize the head at the superior strait; or could not be locked without difficulty, which rendered it useless; and the operator, after a fruitless attempt to apply it, has thrown it by in despair, and condemned the instrument altogether. There can be as little doubt, also, that partiality for the short forceps, which can be used only when the head is at the inferior pelvic strait, has greatly contributed to the infrequency with which they are resorted to by English practitioners. National prejudice has seldom manifested itself more strongly than in the tenacity with which they cling to the short forceps. All the forceps of that country are very much alike, differing only in an endless variety of slight deviations. It is also worthy of remark that the French and German practitioners are nearly unanimous in their preference for the long forceps.

In France, as has been well remarked, they all bear a "family likeness to the forceps of Levret," possessing in greater 'or less degree the second curve, which corresponds to the general axis of the pelvis, and which, with their greater length, renders them suitable for penetrating the parturient passages and seizing the head at the brim, if necessary. Does not this difference account, in part at least, for the greater partiality for the instrument, and the greater frequency of its use on the continent than in England.

Whilst in France and Germany they are resorted to by some as often as every seven, and, by those obstetricians who use them least, as often as once in "250" labors; in England and Ireland only one in six or eight hundred is thought suitable for their application. Thus we find Dr. Seibold, of Berlin, who uses a long instrument, according to the valuable tables furnished by the last American edition of Churchill's Midwifery, had recourse to forceps once in every seven cases—and *craniotomy* only once in 2093 cases. Dr. Collins, who recommends the short forceps, employed it only once in 617 labors, and resorted to the graver operation of perforation once in 141 cases, being nearly 4½ times as often as he used the forceps. These men are among the very first practitioners in their respective countries; and yet we find the celebrated Irish accoucheur resorted to craniotomy 14 times as often as the no less distinguished continental physician' whilst the latter delivered by forceps 8 times as often as the former.

Nor are these by any means rare examples. Drs. Clarke, Ramsbotham, and in short almost all English writers advise that the forceps be used *only* when the head has descended so "that an ear can be felt," &c. deeming it proper to resort to them but once in 700 labors, or thereabouts. Whilst the best French accoucheurs apply the forceps at least three or four times in the same number of cases, and with a corresponding infrequency in the number of cases of embryotomy. Is not this wonderful difference in the frequency of using this instrument to be ascribed in a great measure to the difference in its form as used by these gentlemen? That the highest proportional frequency may not claim imitation, we will admit. But is it not apparent that there may be danger of falling into the opposite extreme and that hyper-caution, and delay, may beget the necessity, often, for a more frequent resort to craniotomy? In this country, we find both the long and short forceps in use, as the practitioner chanced to adopt as his text-book, and guide, a French or an English author.

As the long forceps only can be applied when the head is high in the pelvis, and may be used equally well at the inferior strait, I am inclined to recommend, with most practitioners at least, its exclusive use. By confining himself to one instrument, the operator acquires greater familiarity with it, and becomes more expert in its application. Besides, as the short forceps are in no respect better for seizing the head, even in the inferior strait, they are entirely unnecessary, and motives of economy would induce many persons to dispense with them altogether.

The form of the long forceps, as we find it scattered through the country, varies greatly, as does also its weight. Most of those which I have examined are bungling modifications of Dewees' improvement of Baudelocques', or of Seibold's. Many of them, made by indifferent mechanics, are much more exceptionable than the original patterns. It is not a matter of surprise that most prudent practitioners are disinclined to resort to this degenerate, unmechanical instrument, or that its use should so often be followed by injury to the structures of the mother, or that the operator should be baffled in his efforts to secure the presenting part of the child between its thick and ill shapen blades, and perhaps utterly fail to close and lock them.

It is true, there is no apparent want of variety in the form of this instrument, as may be seen by examining the numerous plates furnished by the modern obstetrical publications. The difficulty seems rather to have been, that each man, conscious of its general defects, has fastened upon some one point, and losing sight of everything else, has strenuously urged the adoption of his fancied or real improvement upon that particular portion of the instrument, attaching this improvement, or modification, very likely to one otherwise so imperfect as to preclude its use. Others, again, have recommended an instrument, the general form of which was admirably adapted to fulfil the end in view, and then rendered its application difficult by leaving, through want of observation, or national prejudice, some important point defective. Thus, Moreau, who gives the cut of an instrument combining in my opinion, more excellencies than any other, unites the blades by means of a "*pivot*," which requires that they should be adjusted with the utmost accuracy before they can be locked, and making it exceedingly difficult of application.

Prof. Hodge has pursued a different course, and one which must, by persevering observation, eventuate in the perfection of the instrument. He places before him all the different forceps in use, and selects from each its peculiar merits, combining them in one. But in an effort to improve his "eclectic forceps", and continue the shafts of the blades in contact, and prevent the vulva from being put upon the stretch unnecessarily early, he has made the angle so acute, when the blades are closed, as to require that its weight should be greatly increased in order to secure the proper power at the distal extremity of the blade. His instrument has also other minor defects which are susceptible of improvement. The blade is too wide more especially at the heel of the fenestrum, the inner edge of the fenestrum is unnecessarily thick, the shoulders of the notch in the female or mortise blade are too abrupt, thus making it difficult to lock, the handles are smooth and cannot be securely grasped, &c. &c. It is however the least exceptionable, in my humble judgment, of any American instrument. Indeed, were the profession generally in possession of an instrument combining as many useful qualities, and as well adapted to administer relief to the suffering female, as the one recommended by the Professor of Obstetrics in the University of Pennsylvania, I should not deem it necessary to obtrude this notice upon the medical public. But, unfortunately, this is by no means the case. Those in use in this section of the country are exceedingly defective. Even Dr. Meigs, of Philadelphia, still, I believe, insists on using the long forceps, without the second or lateral curve, which must seriously impair its usefulness for application at the superior strait, as it cannot conform to the natural curvature in the passages, and must endanger the perineum from pressure.

The instrument which I have used during the last few years is a long forceps, and is considerably curved upon its lateral aspect. It measures in its entire length (*a a* to *b b*), conforming the line measured to the curvature of the blades, 17½ inches. The blades and their shafts to the pivot being about 10, the handles about 7½ inches. The blade (*a* to *d*) is 6½ inches in length, and 7 lines at its narrowest point (*d*), and 1⅞ inches at its broadest point (*e*). The fenestrum is one inch at the widest part (*f*), and gradually diminishes to less than one half of an inch at the heel. The inner or fenestral margin of the blades are ground down so as not to exceed one sixteenth of an inch in thickness, the width (*e* to *f*) being scarcely 5 lines, a
a
g
g
d
d
SIEFFERT
BUFFALO
c
h
h
b
b
a
e
f
d
c
h
b and not exceeding one line in thickness at its periphery (*e*), being considerably thicker in the centre, (midway between *e* and *f*).

The shaft of the blade is scolloped out considerably toward the pivot, upon its inner surface, beyond the termination of the fenestrum.

The points of the blades when the instrument is closed (*a* to *a*) are but 5 or 5½ lines apart, and at the widest point (*g* to *g*) they are 2 inches and 7 lines apart, on the upper or concave surface; whilst on the lower or convex surface, they are slightly more expanded.

The shafts of the blades (from *d* to *c*) approach each other rapidly, but not abruptly.

The blades at the centre of their points (*a*) deviate 3½ inches from the strait line in forming their second or pelvic curve. The entire thickness of the closed instrument at their point of junction (*c*) is less than six lines.

They are united by means of the German notch and button, or screw, which is counter-sunk in the female blade. The edges or shoulders of the mortise, or notch, are rounded, or pared off for four or five lines on either side, so as to incline the pivot to slide into the notch. The mortise is not carried very deeply toward the opposite side of the blade, which would greatly diminish its strength at this point.

The handles diverge in the centre (*h h*) to 1½ inches, and each is expanded or flattened to ¾ of an inch in width at that point, and well roughened on the outer surface, so as to be securely grasped. The points are contracted again, curved and polished, and will separately answer the purpose of blunt hooks. The one encloses a perforator, and the other a sharp hook or crotchet. Each is made oval, and the sheath enveloping it is secured by means of a small transverse screw, which may be removed by the point of a penknife or scissors. The entire instrument is made of the best German or cast steel. Mr. J. Seifirt of this city, a native of Berlin, who makes the instrument very well, prefers the former as being less liable to break.

Here it is perceived we have a very light and graceful instrument of sufficient length to seize the head at the superior strait without difficulty, leaving the lock entirely free from the external organs. The curve is such also as to conform to the direction of the passages, without exerting injurious pressure upon the perineum. The shafts of the blade approximate so as not to distend the vulva before the descent of the head. They incline, however, so gradually as not to diminish their power, as is the case with the instrument of Dr. Hodge. By the politeness of Dr. W. Cary of this city, a pupil of Prof. Hodge's, I am enabled to compare the instruments, and I find that although the forceps of Prof. H. are three ounces heavier, they spring or yield more, being more dilated, by the same amount of force applied at the distal extremity of the blade than those just described.

Besides, the claw or blade of the latter is nearly an inch narrower, and hence it is introduced with much greater facility. It will be found that the concavity of the fenestrum, *levelling* off the inner edges of the blades, will render it better adapted to fit accurately the parietal protuberances, and prevent those salient points from being injured or indented by the sharp angles usually found in this situation. Moreover, this is the widest part of the fœtal head, and the surface to which the fenestrum is ordinarily applied. And if this margin of each blade be two or two and a half lines in thickness, as is the case in many instruments, the pelvic space which will be requisite for delivery, will be three lines less in using one than the other form; or, which is equivalent to the same thing, the amount of compression of the fœtal head must be three lines more in consequence of unnecessary thickness of this edge of the instrument.

One of the difficulties in the application of the forceps consists in uniting the blades, after they have been carried to the requisite height. In the instrument represented, this end is greatly facilitated, slightly lessening the weight at the same time, by cutting away the abrupt shoulders to the mortise, into which the screw easily glides, whenever it gets within these inclined planes.

Again, who ever has been compelled to hold on to well polished round steel handles for any considerable time, will readily appreciate the comfort, as well as sense of security which a roughened and expanded surface must afford. It adds but slightly to the weight of the instrument to increase the length of the handle, and bend it so as to form a blunt hook, and may be a source of considerable convenience. A very good perforator may be inserted into the extremity of one handle, and a sharp hook into the other, and though they may not be of the most approved patterns, they answer very well, should the work of destruction become unavoidable. This arrangement is more important in country than city practice, as one instrument is much more portable as well as more economical than four.

At first I made the end of the handle round and united the perforator, which was of course small and round or triangular, like a common trochar by means of a screw as recommended by Moreau. But by flattening the extremity of the handle, and then securing the shield to the sharp point by a transverse pin or screw, I have been able to obtain a perforator of much better shape, and which, by rotation greatly increases the size of the orifice made in the fœtal skull, and that, too, without increasing its weight. It has been suggested that roughening the centre of the handle must render it liable, when used as a hook or perforator, to irritate the soft parts of the mother. But no operator, I apprehend, would ever use it for this purpose, if smooth, without carrying his finger up beside it. Being careful then to oppose the centre of the roughened side to the hand, would effectually protect the woman from injury, and obviate this objection,

I do not suppose that the instrument as represented is insusceptible of improvement. But it is claimed that it can be used with much greater care and safety than those to be found in the shops of the cutlers in this section of country. It is very light, may be applied at the brim, in the cavity, or at the outlet of the pelvis, by simply varying the direction of the handles. It is less likely to do injury to the child and maternal organs than those in common use, and were it, or some better form than those now in use, generally introduced, much of the repugnance on the part of the Profession to the early employment of this instrument would be overcome; the delay and suffering of the mother would be thereby lessened, with increased safety to her structures, and far fewer children would be subjected to destructive operations. Yours, &c.

BUFFALO, April 15, 1849. JAMES P. WHITE.

## Figures and Tables

**Figure f1:**